# Comparative Evaluation of the Functional and Immunomodulatory Properties of Raw and Spray-Dried Donkey Milk

**DOI:** 10.3390/foods15183256

**Published:** 2026-09-15

**Authors:** Ana-Maria Plotuna, Ionela Hotea, Kalman Imre, Viorel Herman, Ileana Nichita, Ionela Popa, Mihai Pop, Emil Tîrziu

**Affiliations:** 1 Faculty of Veterinary Medicine, University of Life Sciences “King Mihai I”, Calea Aradului, No. 119, 300645 Timisoara, Romania; anamaria.plotuna@usvt.ro (A.-M.P.); kalmanimre@usvt.ro (K.I.); viorelherman@usvt.ro (V.H.); ileananichita@usvt.ro (I.N.); ionela.popa@usvt.ro (I.P.); emiltirziu@usvt.ro (E.T.); 2 Department of Biotechnologies in Pharmaceutical Industry, Faculty of Biotechnologies, University of Agronomic Sciences and Veterinary Medicine of Bucharest (USAMV Bucharest), 59 Mărăști Blvd, Sector 1, 011464 Bucharest, Romania; mihai.pop24@bth.usamv.ro

**Keywords:** donkey milk, spray drying, bioactivity preservation, functional food, rabbit model, immunonutrition, milk processing

## Abstract

Donkey milk contains bioactive components with immunomodulatory potential, but preservation technologies must retain these effects to support its use as a functional food ingredient. This study investigated whether spray drying preserves the in vivo biological functionality of donkey milk. Forty-five female Pannon White rabbits subjected to a standardized vaccine-induced immune challenge were randomly allocated to three groups: one Control group and two groups receiving raw or reconstituted spray-dried donkey milk (2 mL/day; *n* = 15/group) for four weeks. Serum lysozyme and total immunoglobulins were monitored longitudinally, while biochemical, hematological, and integrated multivariate profiles were assessed. Lysozyme increased progressively in both supplemented groups, reaching 29.4% and 33.1% above baseline with raw and spray-dried milk, respectively; cumulative responses were statistically equivalent. Total immunoglobulins recovered earlier after both treatments, reaching 31.31 and 28.92 mg/mL, respectively, versus 23.04 mg/mL in Control; their mean response profiles were closely aligned, although inter-individual variability reduced the precision of the equivalence assessment. Biochemical and hematological assessments revealed no coordinated adverse response to either milk preparation. Moreover, the integrated immune, biochemical, and hematological profiles showed strong concordance between Raw and Spray. Overall, spray-dried donkey milk retained the principal biological responses elicited by raw milk, supporting its potential development as a stable bioactive dairy ingredient.

## 1. Introduction

The practical use of donkey milk as a functional food ingredient is constrained by its limited production, seasonal availability, high perishability, and dependence on refrigerated storage and transport. Transforming the liquid milk into powder could increase its stability, facilitate distribution, and enable its incorporation into specialized foods and nutraceutical formulations. However, these technological advantages are meaningful only if the reconstituted product retains the biological properties of the original milk. Determining whether spray drying preserves these properties is therefore essential for the functional valorization of donkey milk.

Donkey milk has a distinctive nutritional profile characterized by a relatively low fat and casein content, a high whey-to-casein ratio, abundant lactose, and a substantial proportion of unsaturated fatty acids [1,2,3,4,5]. These characteristics contribute to its digestibility and compositional resemblance to human milk and have supported its use in specialized nutrition, particularly for selected children with cow’s milk protein allergy [6,7]. Beyond its nutritional value, donkey milk contains a diverse range of bioactive constituents that may collectively contribute to its functional properties [8,9].

The biological activity of donkey milk is associated with the combined action of its proteins, peptides, oligosaccharides, fatty acids, vitamins, and other components of the milk matrix. Lysozyme is one of its characteristic bioactive proteins and contributes to its antimicrobial potential and interaction with the intestinal environment [10,11]. However, the physiological response to donkey milk may also involve digestion-derived peptides, nutrients, and indirect signaling through the intestinal microbiota and mucosal immune system [12,13,14,15]. Consequently, the biological functionality of donkey milk cannot be predicted solely from the concentration or activity of an individual component.

Production of donkey milk powder offers a practical means of reducing the limitations associated with the raw product [15,16,17]. Spray drying rapidly removes water from atomized milk droplets, producing a low-moisture powder that is more suitable for storage, transport, standardization, reconstitution, and food formulation [16,17,18]. The quality of the resulting powder depends on several interacting parameters, including feed solids, inlet and outlet air temperatures, atomization conditions, airflow, and residence time [16,17,18]. Although the short thermal exposure and evaporative cooling inherent to spray drying can limit heat damage, processing may still modify protein structure, solubility, enzymatic activity, and other quality attributes [18,19,20,21].

Most available research on processed donkey milk has focused on physicochemical composition, microbial stability, powder characteristics, or the retention of selected proteins [19,20,21,22,23,24,25]. These measurements are necessary for characterizing the product but cannot establish whether the reconstituted powder produces biological effects comparable to those of raw milk. Preservation of gross composition does not necessarily demonstrate preservation of functionality, whereas a processing-related alteration in an individual component may not result in an equivalent loss of the biological activity of the complete milk matrix. Controlled in vivo evidence directly comparing raw and reconstituted spray-dried donkey milk remains limited.

Therefore, the present study was planned to evaluate whether spray drying preserved the functional effects of donkey milk after reconstitution. Raw milk and spray-dried milk obtained from the same initial milk pool were administered to rabbits subjected to a controlled immune challenge. Longitudinal serum lysozyme and total immunoglobulin responses were evaluated together with biochemical and hematological variables to characterize immune responses and systemic compatibility. We hypothesized that reconstituted spray-dried donkey milk would elicit biological responses comparable to those induced by raw milk, thereby supporting its potential development as a stable functional food ingredient.

## 2. Materials and Methods

### 2.1. Study Design, Animals and Ethical Approval

A randomized, controlled, parallel-group study was conducted to compare the functional and immunomodulatory effects of raw and spray-dried donkey milk in rabbits subjected to a standardized immune challenge. The experiment was designed to determine whether spray drying preserves the in vivo biological activity of donkey milk following reconstitution through a direct comparison with raw milk under standardized experimental conditions.

A total of 45 clinically healthy, six-week-old female Pannon White rabbits (*Oryctolagus cuniculus*) with a mean initial body weight of 1.27 ± 0.15 kg were obtained from a specialized commercial breeding facility. They were allocated equally to three experimental groups (*n* = 15 per group): a vaccinated control group receiving the basal diet only (Control), a vaccinated group supplemented with raw donkey milk (Raw), and a vaccinated group supplemented with reconstituted spray-dried donkey milk (Spray). The sample size was selected based on the repeated-measures design, experimental feasibility, ethical considerations, and comparable rabbit studies. Personnel responsible for animal allocation and treatment administration were aware of group assignment, whereas laboratory personnel assessing the outcomes were blinded through coded sample identification. Statistical analyses were performed with group identities disclosed. Animal welfare was monitored daily based on general condition, behavior, feed and water intake, mobility, and signs of pain, distress, or disease. No adverse events occurred, and no animals or data points were excluded from the analyses. Each rabbit was individually identified to ensure accurate treatment administration and longitudinal sample collection.

**Figure 1:**
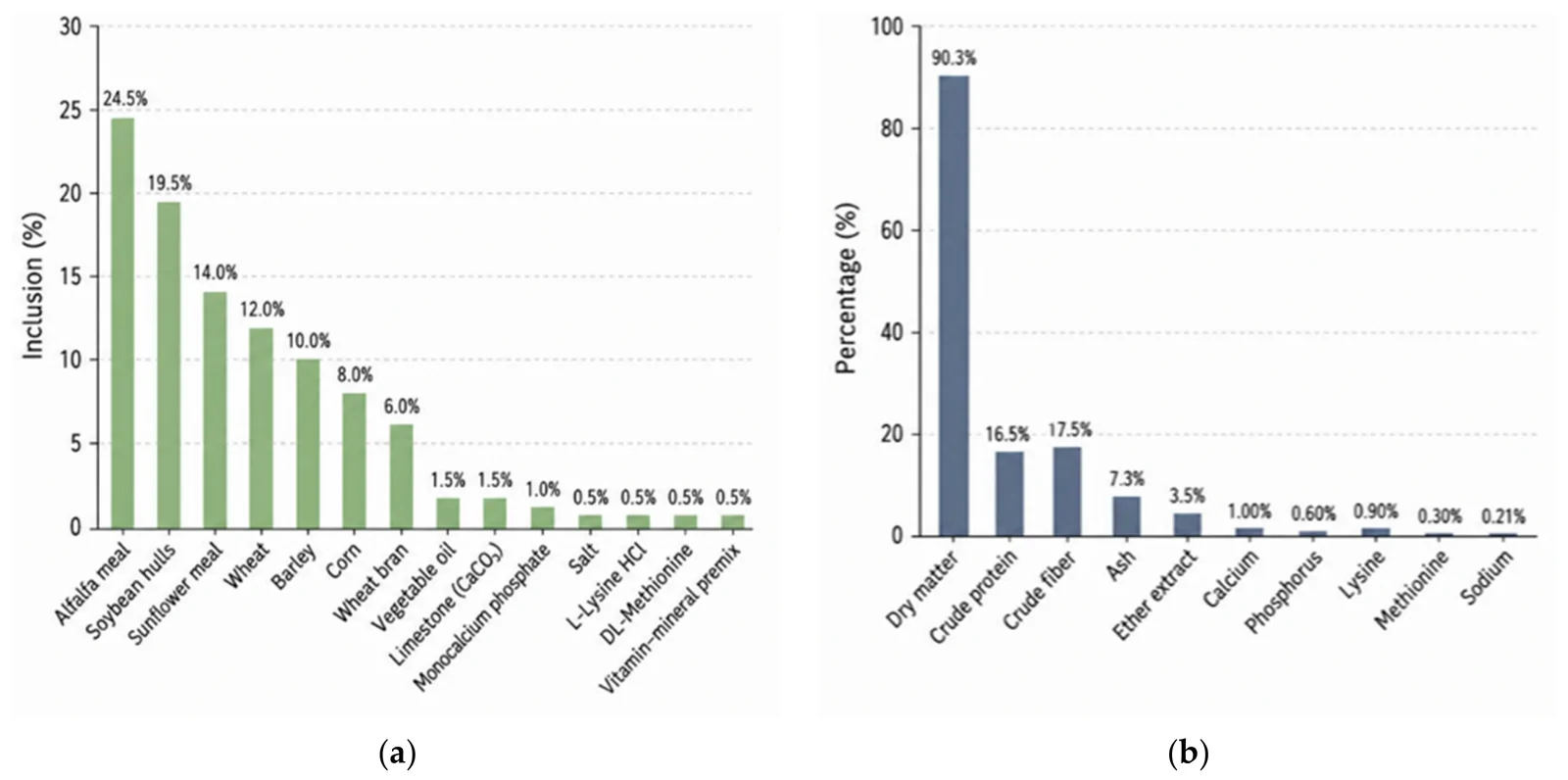
Ingredient and chemical composition of the commercial pelleted diet for rabbits: (**a**) Ingredient inclusion levels, expressed as percentages of the diet on an as-fed basis. (**b**) Analyzed chemical composition, expressed as percentages on a dry-matter basis.

Rabbits were housed individually in galvanized wire-mesh cages equipped with individual feeders and automatic nipple drinkers. Environmental conditions were maintained uniformly throughout the experiment to minimize the influence of external factors on the evaluated outcomes. All animals had ad libitum access to fresh water and the same commercial pelleted diet formulated for growing rabbits (GA Animal Nutrition SRL, Romania; product code GA600745). The basal diet remained unchanged throughout the study, ensuring that donkey milk supplementation was the only dietary variable among the experimental groups. The ingredient composition and analyzed chemical composition of the basal diet are presented in Figure 1.

The experiment comprised five predefined sampling time points (T0–T4), covering the baseline assessment and four consecutive weekly follow-up periods. At T0, blood samples were collected from all rabbits before vaccination and before the initiation of milk supplementation. Immediately after baseline sampling, each animal received a single subcutaneous injection of 0.3 mL of an inactivated vaccine containing Mycoplasma agalactiae strain S/94 (Agalaxin^®^, Romvac, Voluntari, Romania). Vaccination was used exclusively to provide a controlled and uniform immune stimulus, allowing the effects of the two donkey milk preparations on the subsequent innate and humoral immune responses to be evaluated under standardized conditions. Additional blood samples were collected at weekly intervals at T1, T2, T3, and T4 to monitor longitudinal changes in the measured outcomes.

Milk supplementation commenced on the day following vaccination and continued daily until the final sampling point at T4. Rabbits in the Control group received no milk supplementation. Animals in the Raw group received 2 mL of raw donkey milk per day, whereas those in the Spray group received 2 mL of reconstituted spray-dried donkey milk per day. The spray-dried product was reconstituted to correspond to the concentration of the original milk, ensuring comparable intake between the two supplemented groups. Both milk preparations were administered individually using sterile disposable syringes without needles. All administrations were performed orally by the same trained operator at approximately the same time each day to ensure complete individual consumption and minimize procedural variability. The 2 mL/animal/day volume was established during acclimatization as the maximum amount that could be administered completely and reproducibly by oral syringe without spillage. This fixed volume was maintained to enable direct comparison between the two milk preparations rather than a body-weight-adjusted dose–response assessment.

All experimental procedures were conducted in accordance with Directive 2010/63/EU of the European Parliament and of the Council and Romanian Law No. 43/2014 concerning the protection of animals used for scientific purposes [26,27]. The experimental protocol was reviewed and approved by the Ethics Committee of the University of Life Sciences “King Mihai I” from Timișoara, Romania (Approval No. 201/09.03.2023).

### 2.2. Donkey Milk Collection and Processing

Fresh donkey milk was obtained as a single batch from a specialized dairy farm located in Western Romania, maintaining a herd of 75 female donkeys. Milk was collected from 25 clinically healthy lactating jennies, aged 3–14 years and between 30 and 90 days in milk at the time of collection. The animals were maintained under standardized feeding and husbandry conditions. Milk was collected manually under hygienic conditions following routine cleaning and disinfection of the udder. Immediately after collection, it was transported under refrigerated conditions at 4 °C to the University of Life Sciences “King Mihai I” from Timișoara for compositional characterization and processing. Upon arrival at the laboratory, the milk was homogenized and maintained at 4 °C for a maximum of two days. The portion designated for raw milk administration was subsequently divided into individual 2 mL aliquots and stored frozen at −20 °C until use. Individual aliquots were thawed as required for administration, thereby avoiding prolonged refrigerated storage and repeated freeze–thaw cycles.

The chemical composition of the collected milk was determined at the university using a Lactoscope FT-IR milk analyzer (Delta Instruments, Drachten, The Netherlands). The milk contained 8.980 ± 0.396% total solids, 1.578 ± 0.314% protein, 0.323 ± 0.205% fat, 6.387 ± 0.240% lactose, and 0.692 ± 0.048% ash. The lysozyme content was determined using the ELISA method, as we described previously [28]. Its mean lysozyme concentration was 0.9513 ± 0.0270 mg/mL.

Following compositional analysis, the homogenized milk was divided into two portions. The first portion was retained without thermal processing, stored under refrigeration at 4 ± 1 °C until administration, and used to supplement the Raw group, whereas the remaining portion was gently homogenized and processed using a GEA Production Minor spray dryer (GEA Process Engineering A/S, Søborg, Denmark). Spray drying was performed using co-current airflow, with an inlet air temperature of 160 °C and an outlet air temperature of 70 °C. Atomization was achieved using a two-fluid nozzle. The operating conditions were selected to obtain a free-flowing powder while limiting the thermal exposure of heat-sensitive milk constituents. Immediately after drying, the powder was collected and stored at 4 °C in sterile airtight containers protected from light and moisture.

A single spray-dried batch was used throughout the experiment. Immediately before administration, the powder was reconstituted daily with sterile drinking water. The amount of powder and the volume of water were calculated based on the dry matter content of the milk before spray drying, thereby restoring the total solids concentration of the original raw milk. Consequently, equal volumes of raw and reconstituted milk provided equivalent amounts of milk solids.

### 2.3. Blood Sampling and Laboratory Analyses

At each sampling time point (T0–T4), approximately 2 mL of blood was collected from the jugular vein of each rabbit under aseptic conditions [29]. Blood intended for serum analyses was transferred into plain collection tubes and allowed to clot at room temperature for approximately 30 min. Samples were subsequently centrifuged at 3000× *g* for 10 min. The separated serum was divided into aliquots for immunological and biochemical analyses, stored at 4 °C, and analyzed within 24 h of collection. At T4, the collected blood was divided between a plain tube for serum preparation and an EDTA-containing tube for hematological evaluation.

Serum total immunoglobulin concentrations were determined using a rabbit-specific enzyme-linked immunosorbent assay kit (ELISA; MyBioSource Inc., San Diego, CA, USA; Cat. No. MBS731376). Serum lysozyme concentrations were measured using a rabbit-specific ELISA kit from the same manufacturer (Cat. No. MBS2602395). Both assays were performed according to the manufacturer’s instructions. Samples and standards were analyzed in duplicate, and absorbance was measured using a microplate reader at the wavelength specified for each assay. Concentrations were calculated from the corresponding standard curves and expressed as mg/mL for total immunoglobulins and ng/mL for lysozyme.

Serum biochemical analyses were performed using a FUJI DRI-CHEM automated clinical chemistry analyzer (FUJIFILM Corporation, Tokyo, Japan) according to the manufacturer’s instructions. The biochemical profile included alanine aminotransferase (ALT/GPT, U/L), aspartate aminotransferase (AST/GOT, U/L), total bilirubin (TBIL, mg/dL), creatinine (CRE, mg/dL), alkaline phosphatase (ALP, U/L), total cholesterol (TCHO, mg/dL), high-density lipoprotein cholesterol (HDL-C, mg/dL), triglycerides (TG, mg/dL), total protein (TP, g/dL), and albumin (ALB, g/dL).

Hematological analyses were performed at the end of the experimental period (T4). EDTA-treated whole-blood samples were submitted to an accredited veterinary diagnostic laboratory (Synevo Vet, Dumbravita, Romania), where complete blood counts were performed according to the laboratory’s validated procedures. The evaluated erythrocyte parameters included red blood cell count (RBC), hemoglobin concentration (HGB), hematocrit (HCT), mean corpuscular volume (MCV), mean corpuscular hemoglobin (MCH), mean corpuscular hemoglobin concentration (MCHC), red cell distribution width (RDW), and reticulocyte count. Platelet count (PLT) and mean platelet volume (MPV) were also measured. Leukocyte assessment included total white blood cell count (WBC) and differential counts of neutrophils, lymphocytes, monocytes, eosinophils, and basophils.

### 2.4. Statistical Analysis

Statistical analyses were performed using R (version 4.x; R Foundation for Statistical Computing, Vienna, Austria), with statistical significance set at *p* < 0.05. Data are presented as mean ± SEM. Longitudinal immunological and biochemical outcomes were evaluated using linear mixed-effects models that accounted for treatment group, time, their interaction, and repeated measurements within individual rabbits, followed by adjusted pairwise comparisons where appropriate. Hematological variables measured at T4 were analyzed using parametric or non-parametric group-comparison methods according to data distribution and model assumptions. Cumulative longitudinal responses were summarized by the area under the concentration–time curve (AUC), and the biological equivalence of raw and spray-dried donkey milk groups was assessed within an equivalence-testing framework. Equivalence margins were defined as ±10% of the mean AUC of the Raw group and were applied consistently to both immune outcomes; equivalence was concluded when the entire 90% confidence interval for the difference between groups fell within these limits. Multivariate response patterns were explored using principal component analysis and hierarchical clustering. Integrated biological similarity between the two milk preparations was evaluated using standardized biomarker profiles and complementary measures of association, agreement, profile concordance, and correlation-structure similarity.

## 3. Results

### 3.1. Serum Lysozyme Response and Biological Equivalence of Raw and Spray-Dried Donkey Milk

**Figure 2:**
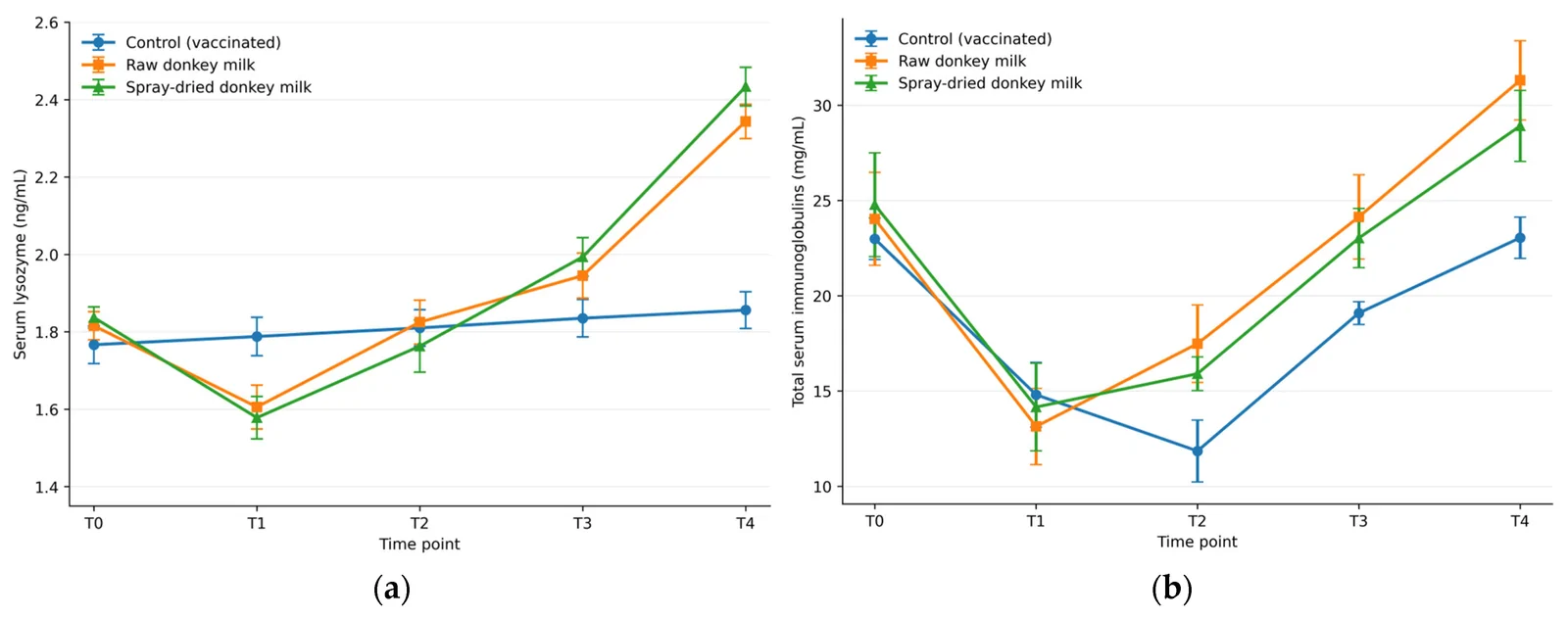
Longitudinal immune responses following controlled immune challenge: (**a**) Serum lysozyme concentrations. (**b**) Total serum immunoglobulin concentrations from baseline (T0) to the end of the study (T4). Values are means ± SEM (*n* = 15 rabbits per group). Milk supplementation began on day 1 after vaccination.

Serum lysozyme concentrations were similar among groups at baseline (Table 1). The linear mixed-effects model identified significant effects of Group (χ^2^ = 6.61, *p* = 0.0368), Time (χ^2^ = 127.16, *p* < 0.001), and the Group × Time interaction (χ^2^ = 90.85, *p* < 0.001). After a transient decrease at T1, lysozyme concentrations increased progressively in both supplemented groups, whereas the Control group remained comparatively stable (Figure 2a). At T4, concentrations reached 2.344 ± 0.044 ng/mL in the Raw group and 2.434 ± 0.050 ng/mL in the Spray group, compared with 1.856 ± 0.048 ng/mL in the Control group (Table 1). The Raw and Spray groups did not differ at T4 (*p* = 0.218), and their final values represented increases of 29.4% and 33.1%, respectively, relative to baseline.

**Figure 3:**
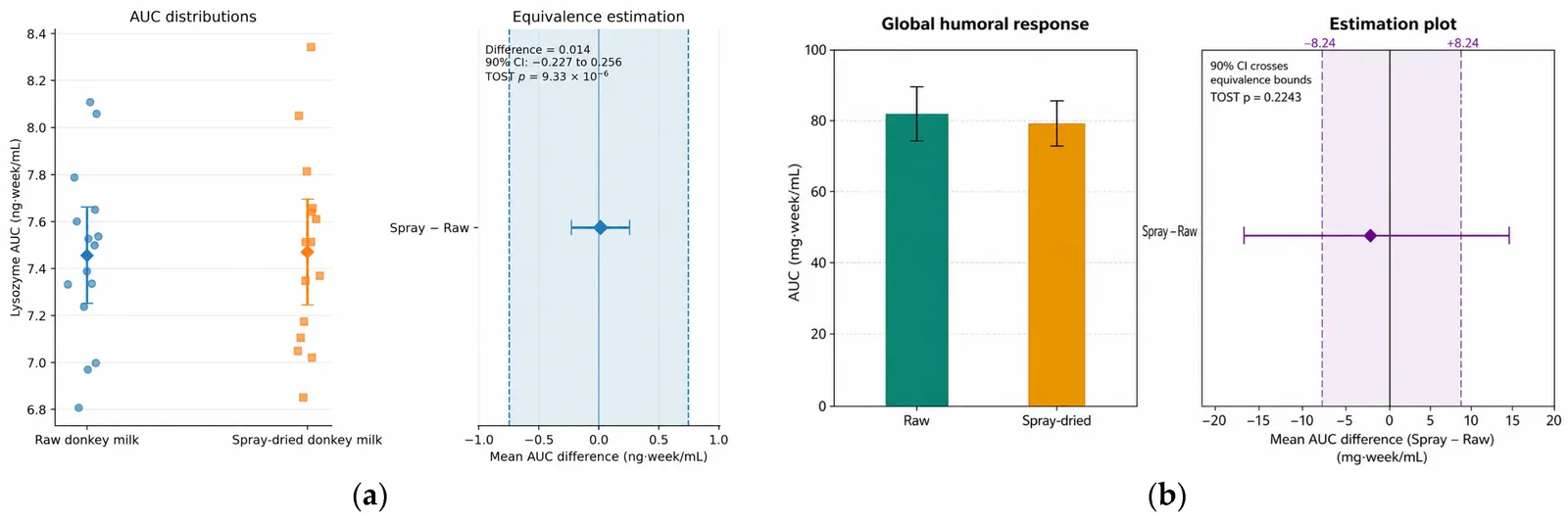
Cumulative immune responses and equivalence assessment for Raw versus Spray-dried donkey milk: (**a**) Lysozyme AUC, for which the 90% confidence interval of the treatment difference remained within the ±10% equivalence limits. (**b**) Total immunoglobulin AUC, for which the 90% confidence interval crossed the equivalence limits. AUC, area under the concentration–time curve; CI, confidence interval; TOST, two one-sided tests.

Cumulative lysozyme responses were virtually identical, with AUC values of 7.456 ± 0.096 ng·week/mL for Raw milk and 7.470 ± 0.105 ng·week/mL for Spray-dried milk. The estimated difference was 0.014 ng·week/mL (90% CI: −0.227 to 0.256). The entire confidence interval was contained within the prespecified ±10% equivalence limits, and TOST confirmed statistical equivalence (*p* = 9.33 × 10^−6^; Figure 3a). Thus, Raw and Spray-dried donkey milk produced comparable longitudinal and cumulative serum lysozyme responses.

### 3.2. Total Serum Immunoglobulin Response to Raw and Spray-Dried Donkey Milk

Baseline total serum immunoglobulin concentrations were similar among groups. Following vaccination, concentrations declined in all groups during the first two weeks and subsequently recovered, with earlier recovery in both milk-supplemented groups (Table 2). The linear mixed-effects model showed significant effects of Group, Time, and the Group × Time interaction (Figure 2b). At T4, concentrations reached 31.309 ± 2.077 mg/mL in the Raw group and 28.917 ± 1.862 mg/mL in the Spray group, compared with 23.043 ± 1.081 mg/mL in the Control group. Raw milk exceeded Control by 8.266 mg/mL (Tukey-adjusted *p* = 0.004), whereas the Spray versus Control comparison approached significance (difference = 5.874 mg/mL, *p* = 0.053). Raw and Spray-dried milk remained comparable at T4 (difference = −2.392 mg/mL, *p* = 0.594), consistent with their closely aligned longitudinal profiles.

Cumulative immunoglobulin responses were numerically similar, with AUC values of 82.44 ± 6.18 mg·week/mL for Raw milk and 79.94 ± 4.19 mg·week/mL for Spray-dried milk. The estimated Spray − Raw difference was −2.50 mg·week/mL (90% CI: −15.26 to 10.26). Accordingly, the two milk preparations showed similar mean humoral-response patterns. However, inter-individual variability resulted in a 90% confidence interval wider than the prespecified equivalence range (±8.24 mg·week/mL; TOST *p* = 0.2243; Figure 3b), limiting the precision of the equivalence assessment.

### 3.3. Serum Biochemical Profile and Metabolic Safety

Baseline biochemical values were similar among groups. Linear mixed-effects models identified Time effects for nine of the ten variables (*p* ≤ 0.0017), whereas total cholesterol remained stable over time (*p* = 0.557). Group effects were restricted to TBIL, ALP, TCHO, HDL-C, and TG (*p* ≤ 0.0146), and Group × Time interactions occurred for nine variables, all except GOT (*p* ≤ 0.0052). These parameter-specific changes did not form a coherent pattern of treatment-associated hepatic, renal, lipid, or protein impairment (Table 3).

**Figure 4:**
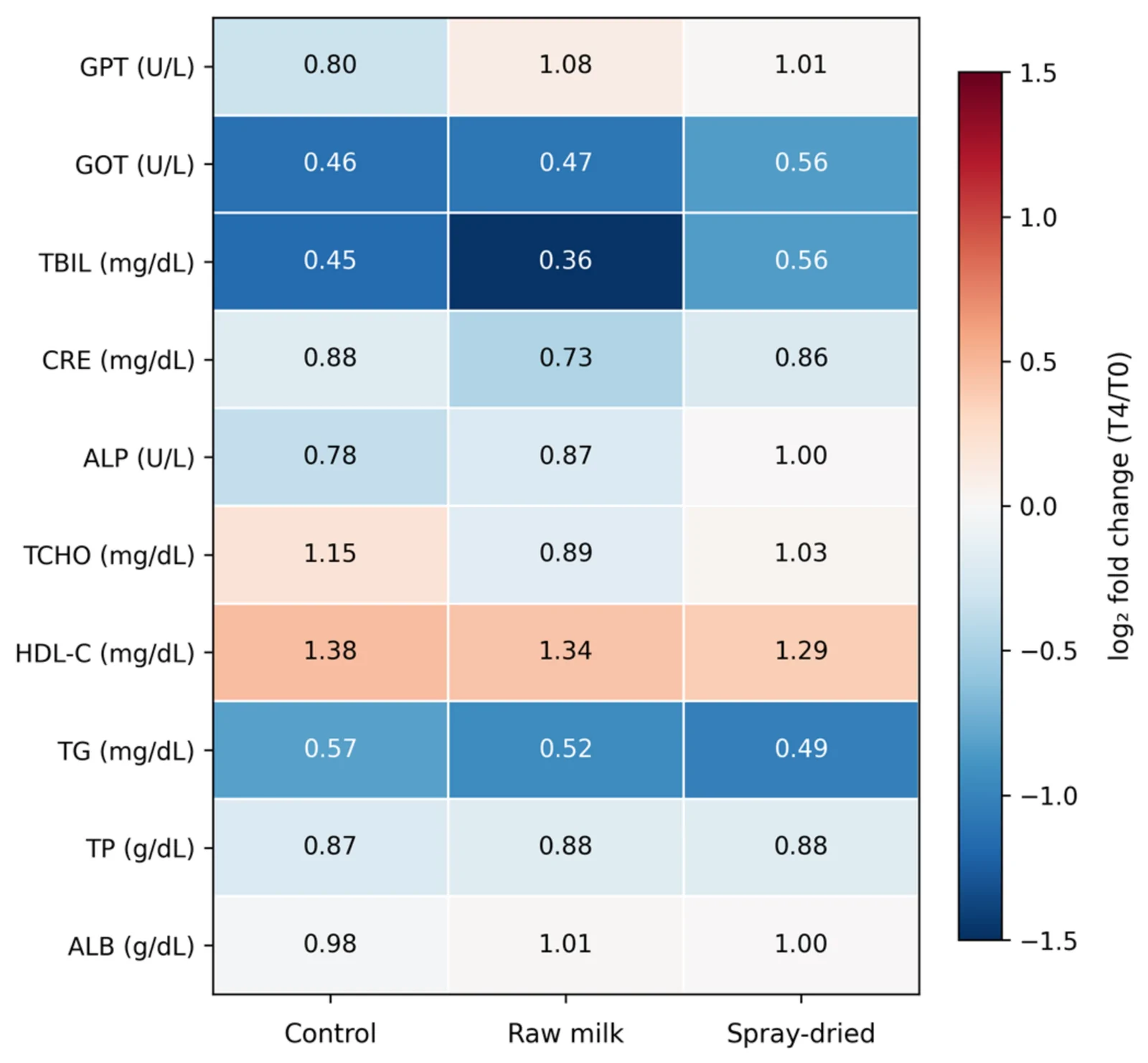
Mean fold changes in serum biochemical parameters at T4 relative to T0. Cells display group mean T4/T0 ratios, while color encodes the log_2_-transformed fold change. Warm shades indicate increases and cool shades indicate decreases (*n* = 15 rabbits per group).

At T4, albumin remained close to baseline in the Raw and Spray groups (fold changes: 1.01 and 1.00, respectively), as did GPT (1.08 and 1.01). GOT, TBIL, CRE, TG, and TP decreased in all groups, while HDL-C increased consistently (1.29–1.38-fold). The decline in ALP was greatest in Control (0.78-fold) and attenuated with Raw milk (0.87-fold), whereas the Spray group remained at baseline (1.00-fold). Total cholesterol changes were modest but heterogeneous (0.89–1.15-fold). Overall, the fold-change profiles were directionally similar between Raw and Spray-dried milk (Figure 4).

### 3.4. Hematological Profile and Leukocyte Redistribution

The hematological evaluation performed at the end of the experimental period (T4) demonstrated that daily supplementation with either raw or spray-dried donkey milk did not adversely affect the overall hematological status of vaccinated rabbits. At T4, RBC, HGB, HCT, MCH, MCHC, PLT, monocytes, and basophils did not differ among groups, indicating preservation of the major erythrocyte, platelet, and leukocyte indices. Variables with significant group differences are summarized in Table 4. MCV was moderately higher in both milk groups than in Control (Raw: 65.55 ± 0.62; Spray: 65.90 ± 0.39; Control: 63.24 ± 0.89 fL; *p* = 0.014), whereas reticulocyte percentages were lower (3.81 ± 0.11% and 3.78 ± 0.08% vs. 5.21 ± 0.38%; *p* = 0.003). RDW and MPV were highest in the Spray group (15.57 ± 0.21%, *p* < 0.001; and 7.93 ± 0.08 fL, *p* = 0.002), but these differences were not accompanied by changes in RBC, HGB, or HCT.

The leukogram showed a modest redistribution of circulating leukocytes. WBC was higher in Spray-dried rabbits than in Control (7.50 ± 0.38 vs. 6.39 ± 0.23 K/µL; *p* = 0.032), with Raw showing an intermediate value. Both milk treatments reduced neutrophil percentages (27.53 ± 0.86% and 27.03 ± 1.75% vs. 33.55 ± 1.22%; *p* = 0.002) and increased lymphocyte percentages (65.47 ± 0.81% and 66.57 ± 1.83% vs. 60.14 ± 1.32%; *p* = 0.004). Eosinophils were higher only in the Raw group (1.95 ± 0.03%; *p* < 0.001). Thus, the principal hematological response was a lymphocyte-predominant shift rather than a generalized hematological alteration.

**Figure 5:**
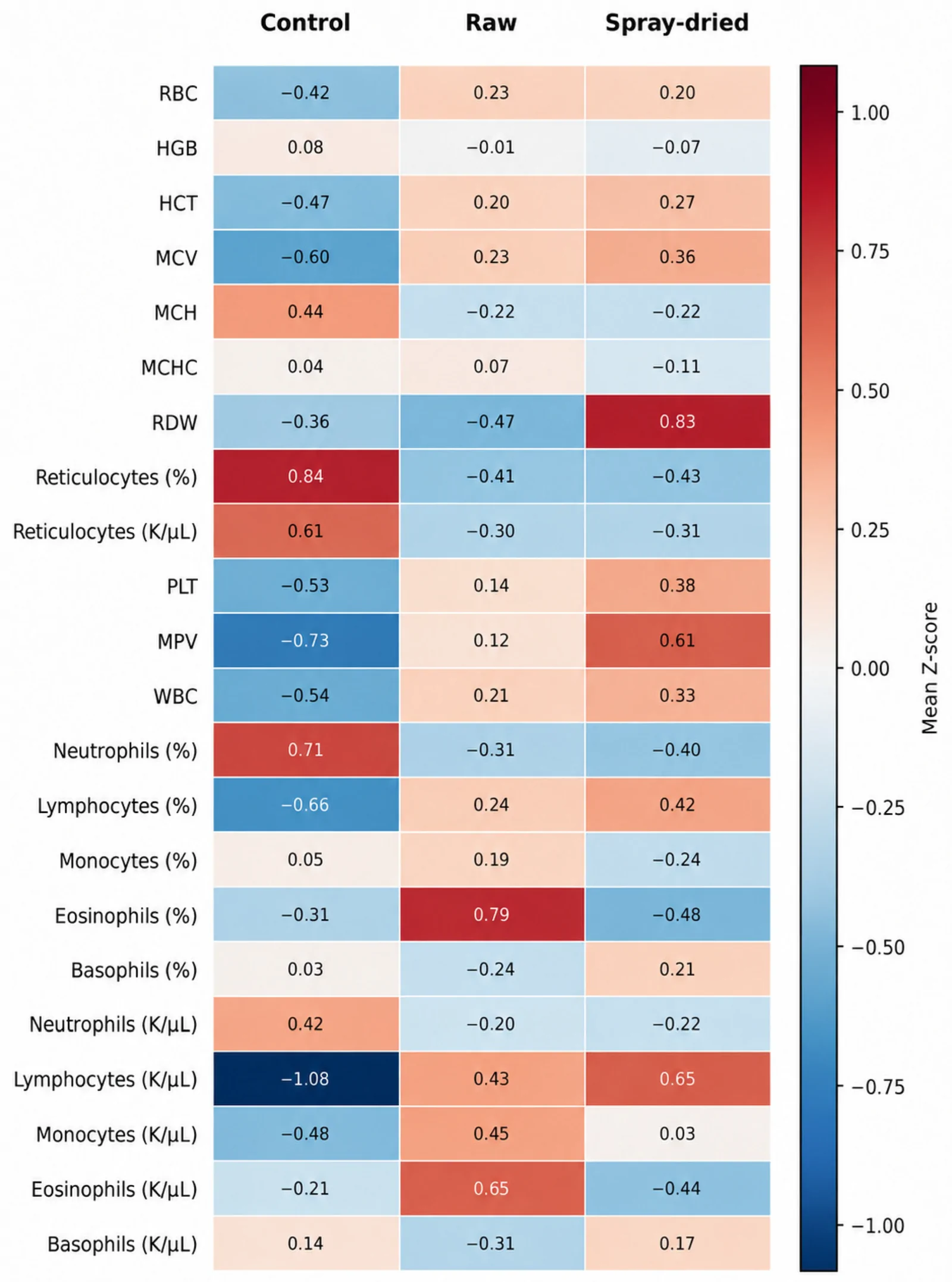
Heatmap of standardized hematological profiles at T4. Cells show group-level mean Z-scores for 22 variables, with red indicating values above and blue indicating values below the overall mean. Hierarchical clustering identified Raw and Spray-dried donkey milk as the most similar treatment profiles (*n* = 15 rabbits per group).

Hierarchical clustering of all 22 standardized variables placed Raw and Spray-dried milk together and separated both from Control (Figure 5). The supplemented groups showed closely aligned Z-score profiles across most variables; the principal divergences involved RDW, MPV, and eosinophils. This multivariate pattern supports a broadly comparable terminal hematological response to Raw and Spray-dried donkey milk, indicating preserved hematopoietic homeostasis following both dietary interventions.

### 3.5. Integrated Biological Similarity Between Raw and Spray-Dried Donkey Milk

**Figure 6:**
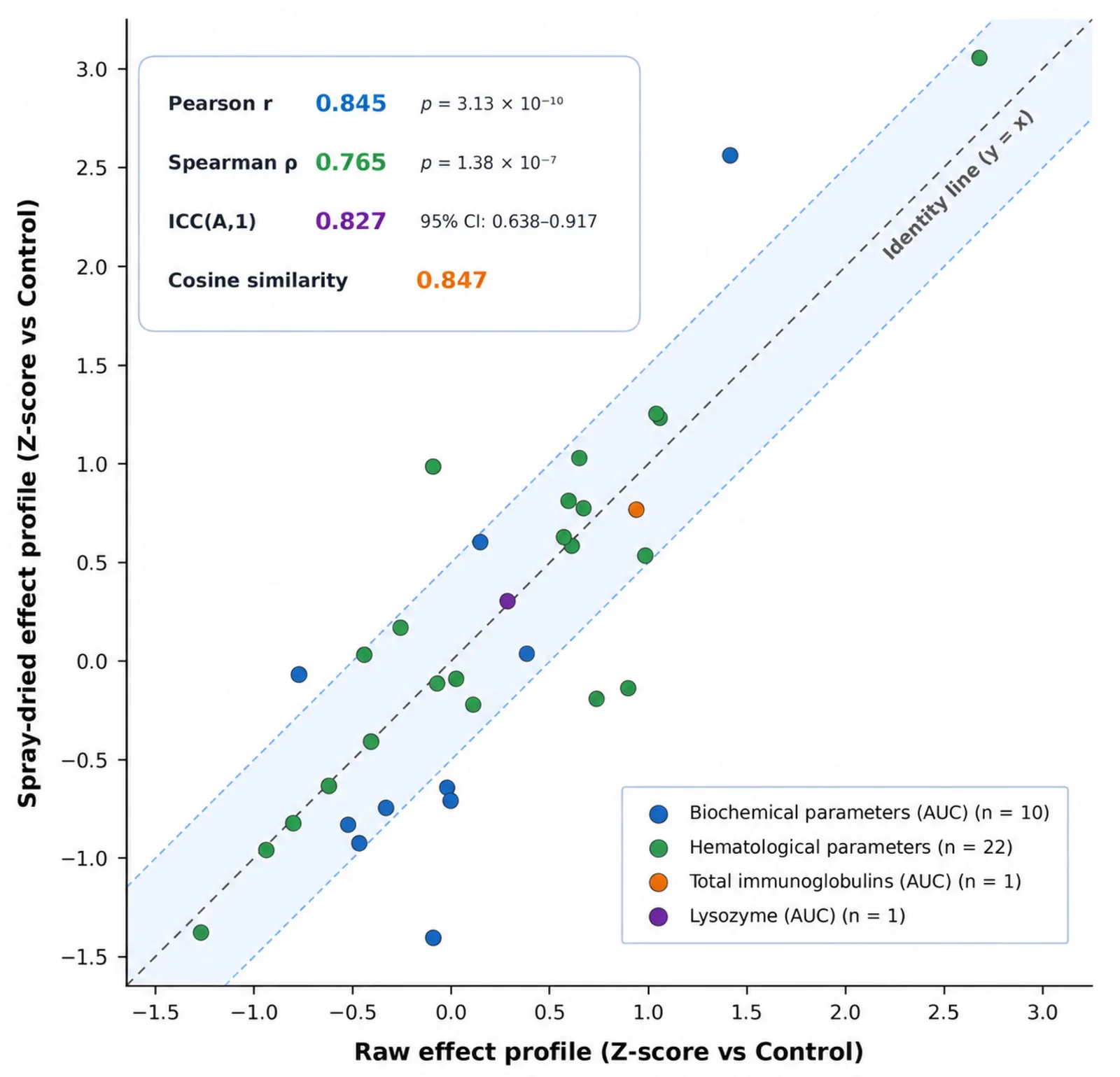
Concordance of the integrated biological effect profiles induced by Raw and Spray-dried donkey milk. Each point represents all analyzed variables expressed as a Z-score relative to Control; the dashed line denotes identity (y = x), and the shaded band denotes close agreement. Colors indicate biochemical (AUC), hematological, and immunological variables.

To evaluate whether spray-drying preserved the overall biological response beyond individual outcomes, the analyzed variables were integrated: AUC values for the serum biochemical parameters, lysozyme, and total immunoglobulins, together with the hematological variables. Responses in the Raw and Spray groups were standardized relative to Control. The resulting effect profiles showed strong concordance (Figure 6), with Pearson’s r = 0.845 (*p* = 3.13 × 10^−10^), Spearman’s ρ = 0.765 (*p* = 1.38 × 10^−7^), ICC(A,1) = 0.827 (95% bootstrap CI: 0.638–0.917), and cosine similarity = 0.847. Most variables were distributed near the identity line, indicating closely aligned directions and magnitudes of response across biochemical, hematological, and immunological domains.

The organization of pairwise biomarker relationships was less strongly conserved than the overall effect profile. Correlation-matrix comparison yielded a Mantel r = 0.279 (*p* = 0.0001; 9999 permutations), an RV coefficient of 0.437, and a mean absolute difference of 0.336 between corresponding correlations, indicating localized reorganization of biomarker associations. Thus, spray-dried milk preserved the principal multivariate biological signature of Raw milk with only limited variation in the relationships among individual biomarkers.

## 4. Discussion

The serum lysozyme response provides the strongest evidence that spray drying preserved an in vivo functional effect of donkey milk. Although baseline concentrations were comparable among groups, the significant Group × Time interaction demonstrated that their subsequent trajectories differed. Following a transient decline at T1, serum lysozyme increased progressively in both supplemented groups, reaching 29.4% and 33.1% above baseline in the Raw and Spray groups, respectively, while remaining comparatively stable in the Control group. The close agreement between the cumulative lysozyme responses, together with the equivalence confirmed by TOST, indicates that reconstituted spray-dried milk retained the innate immune-related effect observed with raw milk.

This response is consistent with the bioactive-protein profile of donkey milk. Previous studies have reported comparatively high lysozyme concentrations, although their magnitude varies with breed, lactation stage, season, animal age, and analytical method [30,31,32]. Donkey milk has demonstrated antimicrobial activity against selected foodborne microorganisms, and both digested and undigested milk retain antimicrobial properties during simulated gastrointestinal digestion [33,34]. The current results extend these predominantly compositional and in vitro observations by demonstrating a sustained systemic response following oral administration.

The increase in serum lysozyme should nevertheless be interpreted as an endogenous host response rather than evidence that intact milk lysozyme entered the circulation. In post-weaning rabbits, dietary proteins are predominantly digested, whereas circulating lysozyme reflects production and release by innate immune cells. Retained lysozyme activity, digestion-derived peptides, other bioactive milk constituents, or indirect signaling through the intestinal microbiota and mucosal immune system may therefore have contributed to the response.

Comparison with processing studies further emphasizes the relevance of the in vivo equivalence. Vincenzetti et al. reported that spray-dried donkey milk retained approximately 58% of the lysozyme activity measured in fresh milk, indicating partial rather than complete preservation [35]. Lysozyme also remained comparatively stable under moderate thermal conditions but declined as treatment intensity increased, while continuous-flow processing produced losses related to the applied thermal load [21,36]. These studies evaluated individual proteins at the product level, whereas the current experiment examined the integrated physiological response after administration. The equivalence between the Raw and Spray groups therefore indicates that partial changes in an individual protein do not necessarily produce a proportional loss of systemic functionality. This interpretation agrees with the findings of Yvon et al., who showed that appropriately thermized donkey milk retained lysozyme activity and reproduced selected gut-barrier and anti-inflammatory effects of raw milk in mice [23]. Collectively, these observations suggest that controlled processing can preserve biologically relevant functions despite some molecular modification.

The preservation of the innate immune response was accompanied by a similar pattern in humoral immunity. Total immunoglobulins initially declined in all groups after vaccination but recovered earlier in the supplemented rabbits. By the end of the experiment, concentrations were higher in both milk groups than in the Control, although statistical significance was reached only for Raw milk, while the Spray comparison approached significance. This distinction should not be interpreted as evidence that only raw milk was biologically active, because the direct Raw–Spray comparison showed no separation and their longitudinal trajectories remained closely aligned. As total rather than vaccine-specific immunoglobulins were measured, these findings describe changes in the overall circulating immunoglobulin pool and cannot be interpreted as evidence of an enhanced vaccine-specific antibody response.

Although direct evidence connecting donkey milk with humoral immunity remains limited, previous studies support the biological plausibility of this effect. Donkey milk oligosaccharides increased antibody titres and antibody-producing cells in mice [37], while donkey milk combined with Lactobacillus rhamnosus enhanced several immune-cell functions, although the contribution of milk could not be isolated from that of the probiotic [38]. Donkey milk consumption has also influenced circulating inflammatory mediators in older adults [39], whereas experimental studies have associated its proteins, peptides, and complete matrix with changes in lymphoid indices, antioxidant status, intestinal microbiota, metabolites, and cytokine production [40,41]. These observations support a potential gut–immune pathway through which donkey milk constituents and microbiota-derived metabolites may influence endogenous immunoglobulin synthesis. A previous rabbit experiment using a multicomponent supplement containing spray-dried donkey milk similarly reported modulation of innate and humoral responses [42]. The current direct comparison extends that work by isolating donkey milk as the intervention and showing that its raw and spray-dried forms elicited closely comparable humoral-response patterns.

The cumulative immunoglobulin responses differed by approximately 3%, supporting similarity between their central estimates. Formal equivalence was not established because inter-individual variability produced a confidence interval wider than the predefined equivalence margins [43]. This result indicates limited precision rather than evidence of a meaningful divergence between preparations. Spray-dried milk therefore closely reproduced the temporal and cumulative mean response to raw milk, although a larger cohort would be required to establish humoral equivalence more definitively.

Importantly, the immunological effects were not accompanied by a coordinated pattern of hepatic, renal, lipid, or protein-metabolism impairment. Although several biochemical variables changed over time, the Raw and Spray groups showed broadly similar profiles, and many changes were also observed in the Control. The absence of concordant alterations across functionally related markers supports physiological adaptation during growth rather than treatment-related organ dysfunction.

This interpretation is particularly relevant in young rabbits, in which biochemical values are influenced by age, breed, nutritional status, handling, and analytical conditions [44,45,46]. The hepatic profile showed no indication of treatment-associated injury because changes in transaminases and bilirubin were shared among groups and albumin remained stable in the supplemented rabbits. Creatinine did not accumulate, and total protein changed similarly across treatments. The ALP trajectory is likewise more plausibly related to skeletal development and bone turnover than to hepatobiliary dysfunction, although specific hepatic and bone markers would be required to confirm this interpretation [44,46].

Triglycerides decreased, and HDL-C increased in all groups, preventing attribution of these changes specifically to donkey milk. Their direction is nevertheless consistent with experimental evidence that donkey milk can influence lipid metabolism, mitochondrial function, and inflammatory regulation [47,48]. In this healthy-rabbit model, however, the results support metabolic compatibility more strongly than a treatment-specific lipid-lowering effect. Clinical studies reporting that donkey milk is tolerated by many children requiring cow-milk-protein exclusion provide additional context for its nutritional compatibility [6]. The current findings therefore indicate short-term biochemical compatibility rather than comprehensive toxicological safety.

This interpretation was reinforced by the terminal hematological profile. Erythrocyte, hemoglobin, hematocrit, platelet, monocyte, and basophil values were comparable among groups, providing no evidence of anemia, impaired oxygen transport, thrombocytopenia, or generalized bone-marrow dysfunction. Comparison with the concurrent Control is essential because rabbit hematological values vary with age, population, environment, and analytical method [49], while postnatal maturation affects both erythrocyte and leukocyte profiles [50].

The modest changes in MCV, reticulocytes, and RDW were not accompanied by alterations in erythrocyte mass or hemoglobinization and therefore did not indicate regenerative anemia or clinically relevant erythrocyte dysfunction [51]. Similarly, the higher MPV in the Spray group occurred without thrombocytopenia or coordinated platelet alterations. Because MPV is sensitive to pre-analytical conditions and cannot independently demonstrate platelet activation, this isolated difference does not suggest impaired primary hemostasis [49,51].

The principal leukocyte response was a shift toward lymphocyte predominance in both supplemented groups, accompanied by lower neutrophil percentages. This pattern contrasts with the relative neutrophilia and lymphopenia characteristic of a rabbit stress leukogram [46] and is consistent with the enhanced lysozyme response and earlier immunoglobulin recovery. It also agrees with experimental evidence that donkey milk constituents can influence lymphocytes, macrophages, natural-killer cells, cytokine production, and gut–immune interactions [37,38,39,40,41].

At the multivariate level, hierarchical clustering grouped Raw and Spray together and separated both from Control, indicating that isolated erythrocyte, platelet, and eosinophil differences did not override their broader hematological similarity. This pattern was consistent with the integrated analysis across the immunological, biochemical, and hematological domains. Following standardization relative to Control, the Raw and Spray effect profiles showed strong linear association, rank concordance, absolute agreement, and vector alignment. Agreement across these complementary metrics indicates that spray-dried milk reproduced both the direction and approximate magnitude of the responses induced by raw milk, supporting preservation of a multisystem biological signature rather than similarity restricted to one biomarker class.

Effect-profile agreement and correlation-matrix similarity nevertheless address different aspects of biological concordance. The former assesses whether the average response of each variable relative to Control was comparable between preparations, whereas the latter examines whether biomarkers covaried similarly among individual animals. The lower similarity between the internal correlation structures therefore does not contradict the strong agreement between the mean effect profiles but suggests that some biomarker relationships were less consistently conserved. Because numerous correlations were estimated from a relatively small cohort, these network-level findings should remain exploratory.

This integrated perspective addresses an important gap in donkey milk research. Preservation technologies should be evaluated not only according to powder characteristics and retention of selected compounds but also according to the biological functionality of the reconstituted product [15]. Spray drying can improve the solubility, storage, transport, and standardization of donkey milk powder [18], although excessive thermal exposure may modify whey-protein structure and reduce the activity of heat-sensitive constituents [52]. Processing intensity may also affect the anti-inflammatory, antioxidant, microbial, and metabolic effects of donkey milk proteins [53]. Under the conditions tested here, however, these potential molecular modifications did not generate a fundamentally different systemic response from that elicited by raw milk.

Such preservation is plausible because the functionality of donkey milk reflects interactions among proteins, oligosaccharides, bioactive peptides, micronutrients, and unsaturated fatty acids rather than dependence on a single heat-sensitive molecule [54]. Residual intact proteins, digestion-derived peptides, protection provided by the milk matrix, and functional redundancy among its constituents may all have contributed to the comparable responses. Direct compositional, proteomic, and functional characterization of the raw and spray-dried preparations would nevertheless be required to determine which components were retained, modified, or functionally compensated after drying.

Several features strengthen the interpretation of the study. Both preparations originated from the same initial milk pool, minimizing compositional variability and isolating processing as the principal experimental contrast. Animals of the same breed, sex, and age were maintained under standardized conditions and subjected to an identical vaccination schedule, while the concurrent Control accounted for temporal changes related to growth and experimental handling. Repeated immunological measurements characterized response dynamics, and the simultaneous assessment of biochemical and hematological outcomes provided a broader evaluation of compatibility.

The analytical strategy was also suited to the central research question. Mixed-effects models accounted for repeated measurements, AUC summarized cumulative responses, and TOST evaluated equivalence directly rather than inferring similarity from a nonsignificant difference. The combination of endpoint-specific and multivariate analyses enabled processing effects to be assessed at both individual-biomarker and whole-profile levels. The convergence of lysozyme equivalence, closely aligned immunoglobulin trajectories, the absence of a coordinated adverse biochemical or hematological pattern, and strong integrated agreement reinforces the internal coherence of the findings.

The results should nevertheless be interpreted within the limitations of a preclinical study conducted in young female Pannon White rabbits. The sample size reduced the precision of the immunoglobulin equivalence analysis and the stability of the exploratory correlation networks. Only one dose, one spray-drying protocol, and one production batch were evaluated over a relatively short period, precluding conclusions about dose dependence, long-term intake, batch reproducibility, or alternative processing conditions. Hematology was measured only at the terminal time point, preventing longitudinal assessment, and its greater representation within the integrated dataset increased its contribution to the global similarity estimates.

Overall, the endpoint-specific and integrated analyses indicate that the spray-drying protocol used in this study preserved the dominant systemic response pattern induced by raw donkey milk. The agreement across innate and humoral immune responses, biochemical compatibility, hematological homeostasis, and the integrated effect profiles supports the potential of reconstituted spray-dried donkey milk as a stable functional ingredient while identifying the product-level and mechanistic questions that require further investigation.

## 5. Conclusions

Under the conditions tested, spray-dried and raw donkey milk produced comparable biological responses in vaccinated rabbits. Formal equivalence of the cumulative serum lysozyme response demonstrated that the spray-dried milk preparation elicited an innate immune-related response comparable to that observed with raw donkey milk. This finding was further supported by closely aligned immunoglobulin trajectories, the absence of treatment-related biochemical or hematological disturbances, and strong concordance across the integrated systemic response profiles. Although both preparations were obtained from the same initial milk pool, the present study did not include a direct compositional comparison between raw and spray-dried donkey milk; therefore, the observed similarity in biological responses cannot be interpreted as evidence that the original milk composition or all functional properties were preserved following spray drying. Nevertheless, the comparable responses observed for the biological parameters evaluated provide a preclinical basis for further investigation of spray-dried donkey milk as a stable ingredient for specialized food and immunonutritional applications. Future studies integrating detailed compositional analyses with biological assessments are warranted to determine the extent to which specific bioactive components and functional properties are retained following spray drying. Further validation using donkey milk powder produced under commercial-scale spray-drying conditions, including conventional concentration and preheating steps, would also be valuable to determine whether the biological responses observed under the experimental processing conditions are maintained following industrial-scale production.

## Figures and Tables

**Table 1:** Temporal changes in serum lysozyme concentrations (ng/mL) in rabbits.

Time	Control	Raw Donkey Milk	Spray-Dried Donkey Milk	Raw vs. Spray (*p*)
T0	1.767 ± 0.049	1.816 ± 0.036	1.837 ± 0.027	0.577
T1	1.788 ± 0.050	1.606 ± 0.057	1.578 ± 0.055	0.812
T2	1.810 ± 0.047	1.825 ± 0.057	1.763 ± 0.067	0.643
T3	1.835 ± 0.048	1.945 ± 0.058	1.994 ± 0.050	0.681
T4	1.856 ± 0.048	2.344 ± 0.044	2.434 ± 0.050	0.218

Values are presented as mean ± SEM (*n* = 15 rabbits per group).

**Table 2:** Temporal changes in serum immunoglobulin concentrations (mg/mL) in rabbits.

Time	Control	Raw Donkey Milk	Spray-Dried Donkey Milk	Raw vs. Spray (*p*)
T0	22.990 ± 1.089	24.037 ± 2.438	24.782 ± 2.723	0.969
T1	14.804 ± 1.698	13.139 ± 1.998	14.158 ± 2.298	0.932
T2	11.851 ± 1.622	17.485 ± 2.036	15.908 ± 0.884	0.763
T3	19.092 ± 0.596	24.141 ± 2.212	23.027 ± 1.555	0.875
T4	23.043 ± 1.081	31.309 ± 2.077	28.917 ± 1.862	0.594

Values are presented as mean ± SEM (*n* = 15 rabbits per group).

**Table 3:** Baseline (T0) and terminal (T4) serum biochemical parameters in rabbits.

Parameter	Control T0 → T4	Raw T0 → T4	Spray-Dried T0 → T4
GPT (U/L)	32.13 ± 1.65 → 27.57 ± 1.56	33.60 ± 1.49 → 35.71 ± 1.96	35.73 ± 2.11 → 32.86 ± 1.72
GOT (U/L)	32.27 ± 2.13 → 15.43 ± 1.10	33.27 ± 1.77 → 14.64 ± 0.61	29.87 ± 1.86 → 15.79 ± 0.96
TBIL (mg/dL)	1.33 ± 0.09 → 0.56 ± 0.04	1.85 ± 0.11 → 0.61 ± 0.05	2.00 ± 0.23 → 0.89 ± 0.11
CRE (mg/dL)	0.76 ± 0.03 → 0.67 ± 0.04	0.82 ± 0.02 → 0.62 ± 0.02	0.75 ± 0.02 → 0.64 ± 0.02
ALP (U/L)	506.60 ± 16.04 → 368.14 ± 14.91	471.27 ± 10.04 → 418.21 ± 11.96	436.73 ± 24.03 → 416.71 ± 21.39
TCHO (mg/dL)	52.60 ± 2.44 → 57.50 ± 3.84	61.87 ± 3.87 → 56.21 ± 3.47	75.40 ± 7.08 → 69.50 ± 5.02
HDL-C (mg/dL)	18.87 ± 1.21 → 23.00 ± 1.65	19.87 ± 0.90 → 27.79 ± 1.42	25.73 ± 1.69 → 31.29 ± 1.33
TG (mg/dL)	91.27 ± 5.61 → 54.86 ± 6.60	120.87 ± 14.87 → 59.86 ± 5.08	111.60 ± 6.99 → 50.36 ± 3.57
TP (g/dL)	6.06 ± 0.15 → 5.46 ± 0.18	6.00 ± 0.39 → 5.83 ± 0.21	6.63 ± 0.13 → 5.96 ± 0.22
ALB (g/dL)	5.63 ± 0.10 → 5.40 ± 0.11	5.75 ± 0.11 → 5.59 ± 0.08	5.90 ± 0.12 → 5.72 ± 0.10

Values are means ± SEM (*n* = 15 rabbits per group); arrows indicate T0 → T4. GPT, glutamate pyruvate transaminase; GOT, glutamate oxaloacetate transaminase; TBIL, total bilirubin; CRE, creatinine; ALP, alkaline phosphatase; TCHO, total cholesterol; HDL-C, high-density lipoprotein cholesterol; TG, triglycerides; TP, total protein; ALB, albumin.

**Table 4:** Hematological variables showing significant differences among groups at T4.

Parameter	Control	Raw	Spray-Dried	*p*-Value
MCV (fL)	63.24 ± 0.89	65.55 ± 0.62	65.90 ± 0.39	0.014
RDW (%)	14.00 ± 0.41	13.85 ± 0.16	15.57 ± 0.21	<0.001
Reticulocytes (%)	5.21 ± 0.38	3.81 ± 0.11	3.78 ± 0.08	0.003
MPV (fL)	6.85 ± 0.27	7.53 ± 0.12	7.93 ± 0.08	0.002
WBC (K/µL)	6.39 ± 0.23	7.34 ± 0.30	7.50 ± 0.38	0.032
Neutrophils (%)	33.55 ± 1.22	27.53 ± 0.86	27.03 ± 1.75	0.002
Lymphocytes (%)	60.14 ± 1.32	65.47 ± 0.81	66.57 ± 1.83	0.004
Eosinophils (%)	1.39 ± 0.16	1.95 ± 0.03	1.30 ± 0.10	<0.001

Values are means ± SEM (*n* = 15 rabbits per group). Significant difference was set at *p* < 0.05. MCV, mean corpuscular volume; RDW, red cell distribution width; MPV, mean platelet volume; WBC, white blood cell count.

## Data Availability

The original contributions presented in this study are included in the article. Further inquiries can be directed to the corresponding author.
